# Supplementary material for: Modeling the formation of a worldwide health network fighting TBC: Key drivers in policy, management and governance in developing countries and global health institutions
Source: PLoS One. 2025 Aug 21;20(8):e0330538. doi: 10.1371/journal.pone.0330538 (PMC12370072; doi:10.1371/journal.pone.0330538)
Supplement: S1 File — (PDF) [file pone.0330538.s001.pdf]

# Supplementary Materials of the manuscript: Modeling the formation of a worldwide health network fighting TBC: key drivers in policy, management and governance in developing countries and global health institutions

## S1 Introduction

This supplementary materials discusses more in detail the equations, and Monte Carlo algorithms behind the experiments on the link formation of a global health network fighting TBC.

## ML classification

In this section, we present the application of machine learning classification methodology to categorize nodes within the original X (formerly Twitter) network of TBC actors (Lopreite et al. 2021) . Our goal was to identify nodes with specific roles: a) institutions, b) donor NGOs, c) receiver NGOs, and d) others.

The original dataset consists of the following fields: URL of the website: This identifies the organization's or institution's website.

User description: A brief self-provided description available to every user on X (formerly Twitter).

Location: Typically the country of the headquarter for NGOs or institutions. This field allows for differentiation between developing countries with high TBC incidence and developed countries with low TBC incidence and high income.

From the user description (field 2), we applied a TF-IDF transformation (with 200 word features and n-grams limited to 2) to generate a sparse matrix suitable for the machine learning classifier. The website URL (field 1) was used to create a binary filter: "institution" or "non-institution." Specifically, the presence of ".gov," ".int," ".europa," and/or "UN" in the URL was used to identify (a) American official government institutions (e.g., CDC Center for Disease Control) or European agencies, and (b) UN agencies (e.g., UNHCR.org). Finally, the location information help us in distinguishing countries with high

| | |
|---|---|
| Donor | 1368 |
| Receiver | 289 |
| Other | 93 |
| Institution | 88 |

Table 1: Node classification for the TBC X network

TBC incidence from those with low incidence, acknowledging TBC as primarily a "low-income" disease.

We used a Random Forest classifier for this task. Given the significant class imbalance within our dataset, we employed the classifier's "balanced" mode, which applies weights to each class during the training phase (for reference, see the scikit-learn Random Forest documentation `https://scikit-learn.org/stable/modules/generated/sklearn.ensemble.RandomForestClassifier.html`). The 80/20 train/test split is applied with CV=10 (10 folds). Metrics such as the the F1 score are averaged on 10 realizations.

The class distribution from the original TBC network model (Lopreite et al. 2021) is as follows:

Notably, potential donors constitute approximately 77% of the total nodes in the dataset. Despite this class imbalance, the Random Forest model achieved a F1 average score of 0.94. This score was obtained after careful hyperparameter tuning and by applying the aforementioned "balanced" weights for class rebalancing during training. As detailed in the confusion matrix plot, the model performs better in identifying receivers (100% correct answer) and donors (96% correct answer). Performance for the institution class is acceptable, with 82% of objects correctly identified, while the "others" class shows a sufficient performance of 57% correct predictions.

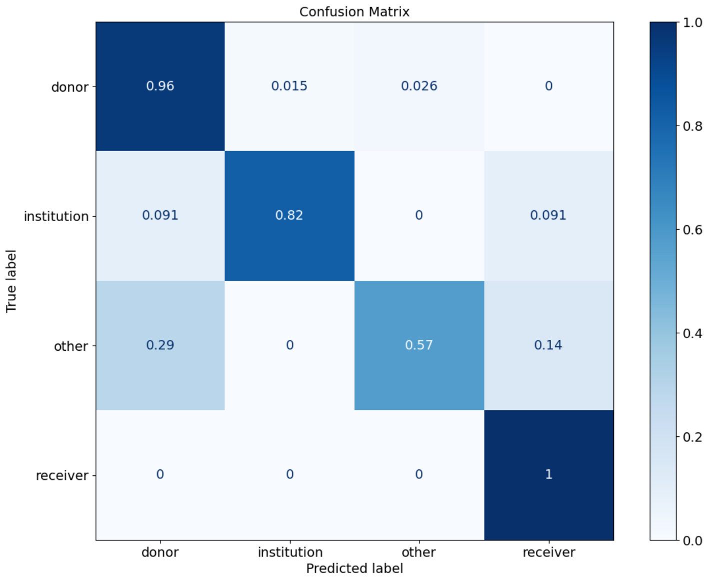


Figure 1: Confusion matrix for the TBC classes (donors, receivers, institutions, other) after Random Forest Classification
