# Supplementary material for: Modeling the formation of a worldwide health network fighting TBC: Key drivers in policy, management and governance in developing countries and global health institutions
Source: PLoS One. 2025 Aug 21;20(8):e0330538. doi: 10.1371/journal.pone.0330538 (PMC12370072; doi:10.1371/journal.pone.0330538)
Supplement: Attachment — Submitted filename: referee_report.docx.pdf [file pone.0330538.s002.pdf]

**Authors' Response to Comments of REVIEWERS**

"Modeling the formation of a worldwide health network fighting TBC: key drivers in policy, management and governance in developing countries and global health institutions"
(PONE-D-25-26747)

PLEASE NOTE:

We would like to thank the anonymous referees for their insightful comments and suggestions, which have helped to improve the paper. The amended version of the paper has been revised along the lines suggested by the referees. In the attachment you will find two versions in Word format of the updated article: one with revisions made explicit and another clean for easier reading. In addition, a summary and explanation of the changes are provided in the table below: comments of the referees are on the left side of the table; our responses are on the right side.

| COMMENTS | RESPONSES |
| --- | --- |
| **Reviewer #2:** In the manuscript, the authors introduce a network formation model to identify the drivers of the health network in the fight against TBC. Further, they investigate how social media popularity, economic size, and disease incidence affect network growth.<br><br>I enjoyed reading the study and found their contribution to be interesting. However, I believe the manuscript could benefit from further clarification regarding the proposed methodology, as well as additional experimental details. Below, I outline my main points of concern. | |

| | |
|---|---|
| **ML model for user labelling**<br><br>In Introduction and Methods, the authors mention that, starting from an X friendship network collected in prior work, they employ a Random Forest classifier to categorize users into four classes (institutions, donors, receivers, others), achieving an 85% accuracy. However, some important details are missing from the presentation. | We thank the reviewer for this important comment and fully agree with the suggestion. In the revised manuscript, we have added the discussion relative to the points (**1**), (**2**), (**3**) as follows: |
| **1.** What features was the model trained on? Were there any pre-processing steps involved? | **1.** *The ML model has been trained on the following features.*<br>*1) Binary country location (i.e. developed vs developing country).*<br>*2) Internet domain of the node (.gov, and .int are clearly referring to institutions, together with references to UN agencies)*<br>*3) Twitter user description: they clearly report if a user is an institution or a charity or an individual (doctor / expert). The user description is treated as a corpus and the ordinary TfIDF treatment applied.*<br>*Regarding the pre-processing, the feature "Internet domain of the done" is obtained by regular expression elaboration of web domain names to get domain suffixes (.gov, .eu, .com etc.) , and the presence of reference to UN agencies (i.e. unhcr ) or WHO local agencies.*<br><br>*Classical text processing for English is applied (stop-words removal, n-gram limited to 2 words, and tdidf max features = 200). The ML classifier is a Random Forest where a simple hyperparameter optimization has been carried out with a grid-search (working on number of* |

| | |
|---|---|
| | *estimators, and max leaf depth). The classifier has been used in "balanced mode", i.e. it applies weights to classes during the training phase.*<br>(**Introduction and Methods**). |
| **2.** How balanced are the classes? Unless classes are very balanced, the F1 score would be a more representative metric. Even better, the authors could display a confusion matrix that compares true and predicted classes. | **2.** *The classes within our dataset are significantly unbalanced as shown by the values predicted by the Machine Learning classifier from the original TBC network model (Lopreite et al., 2021):*<br>*Donor 1368*<br>*institution 88*<br>*other 93*<br>*receiver 289*<br><br>*Notably, the potential donors are approximately 77% of the total number of nodes in the dataset.* ( **Methods**).<br><br>*Despite this class imbalance, the Random Forest model achieved an F1 score of 0.94. This score was obtained after hyperparameter tuning and by applying "balanced" weights for class re-balancing during training. For a complete analysis, we provide the confusion matrix, which compares true and predicted classes, in the Supplementary Materials.*<br>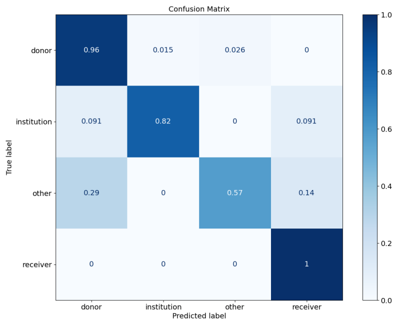<br><br>*As shown in the Confusion matrix plot the model performs better in identifying* ***receivers*** *(100% accuracy) and* ***donors*** *(96% accuracy). Performance for the* |

| | |
|---|---|
| | ***institution*** *class is acceptable, with 82% of objects correctly identified, while the* ***"others"*** *class shows a sufficient performance of 57% correct predictions.* (**Supplementary Materials**) |
| **3.** Is the reported performance the average across CV samples (how many?) or on a separate test set? In the latter case, how large (in terms of number of accounts) is the training/validation set vs the test set? | **3.** *We employed a standard 80/20 training/test split, performing 10-fold cross-validation (CV=10). Model performance, and F1 score has been averaged across these folds.* (**Methods**) |
| **Network formation model** | We thank the reviewer for the helpful comments. To address the points (**1**), (**2**), (**3**), (**4**). (**5**) we have clarified as follows: |
| **1.** The authors mention that the number of followers and "economic size" are both characterized by skewed distributions. This is reasonable. However, plotting the two distributions with a fit line or a goodness-of-fit test would better motivate the use of the exponential distribution. | **1.**We've added a QQ-plot (Figure 1 left-right) with exponential fitting line of the distributions to the "**Methods**" section, under the subsection "**Modeling network formation**" |
| **2.** Given that popularity and economic size are on different scales (see the quantiles in Table 1), won't the average popularity be typically smaller than the average economic size (Eq (2) in SI)? Is this something that may affect the results, or have the authors taken it into account? In | **2.** We added to the main manuscript the following part: *Crucially, our network model works precisely because of the "different scales" of popularity and economic size. This distinction allows us to demonstrate that* ***economic size*** *is the primary driver for* |

| | |
|---|---|
| the Discussion, the authors say that node formation is likely driven by economic size, but unless some form of standardization is applied, this may just be a matter of scale. | *connections in the TBC X social network.*<br><br>*For NGOs nodes we assumed the "economic size" relevant in terms of potential funding opportunities. This economic interest is directly reflected into how nodes form connections within the network model. Popularity, instead, plays a significant role only for entities of similar economic size and similar operational roles. In fact, our analysis indicates that popularity only becomes the dominant factor when the economic interest is weak. The network model's logic is designed to capture this dynamic when a link is formed.*<br><br>*In summary, it is the different scale of these two variables (popularity and economic size) that explains the link formation in the original network.*<br><br>*Finally, we notice that on a platform like X (formerly Twitter), economic size is not explicitly attached to user nodes. A significant aspect of our contribution is filling this data gap: prior to our work with d-portal in collecting financial exchanges among NGOs, there was no accessible information regarding the characteristic economic size or distribution of these organizations. This also brings into question the prevailing hypothesis that non-profit organizations and their economic size necessarily scales according to a power law or fat tail.*<br><br>(**Results**) |
| **3.** I think a clearer definition of the probability P (Eq (2) in SI) should be provided. | **3.** We've clarified the definition and included the formula in the main manuscript. (**Results**) |

<table>
<tr>
<td>

**4.** I'm not confident that comparing the degree distributions is the best way to validate the simulation outcomes. The authors themselves acknowledge that "different network formation models can eventually reproduce the same degree distribution". For instance, as an additional metric, the authors could compute the Jaccard similarity of the two edge sets.

</td>
<td>

**4.** We've addressed this point in the revised manuscript by incorporating the clarifications of **Part (2)**. **Part (1)** serves as our direct reply to the referee here.

**Part (1): for the reviewer**

*The simulation procedure generates numerous random replicas of the original network in each Monte Carlo run, but it does not preserve the exact economic or popularity size of the nodes. Instead, these two quantities are randomly generated and assigned to the nodes at every step.*

*Consequently, an exact overlap measure like the Jaccard index, which relies on counting common objects between sets, is not suitable here. Even if we fix parameters, the economic and popularity sizes in each run are random and only statistically similar. This makes Jaccard computation problematic, as it would require discretizing these continuous values, which presents significant challenges for accurate representation. For these reasons, we did not use the Jaccard index. However, in response to the reviewer's suggestion, we have incorporated an alternative local measure.*

**Part (2): in the main manuscript (Discussion)**

*To enhance our analysis, we've augmented the initial degree distribution analysis with the local clustering coefficient. This node-level metric quantifies a node's propensity to form triangles, indicating the presence of strong social ties. It offers a more in-depth understanding than a simple node degree distribution by exploring the connectivity among a node's immediate neighbors.*

*The simulated distribution of local clustering is similar to the actual one (see Figure 2 C bottom plot), successfully*

</td>
</tr>
</table>

| | |
|---|---|
| | *reconstructing the correct density. However, the true network contains additional information our model can't fully capture.*<br><br>*For example, nodes representing the UN agencies operate internationally and likely exhibit different behavior compared to institutions specifically focused on health, such as the CDC or WHO. The discrepancies observed between the simulated and actual network clustering are likely due to the existence of internal, national, regional, or even role-based network communities.*<br><br>*It is important to notice that our model is intentionally conservative. It focuses solely on the interplay between donors, receivers, and disease levels, deliberately omitting information related to geography, communities, or specific node roles (beyond the primary classification of donors, receivers, institutions, and others).* |
| **5.** In Fig. 1, I don't understand why two curves (economics prevailing or popularity prevailing) are shown instead of just one. Isn't it just the value of lambda that determines which of the two is prevailing? | **5.** This point is not included in the manuscript; it is addressed solely in our response to the reviewer.<br><br>*Fig. 2 (A) displays three curves, illustrating the proportion of links formed due to popularity, economic factors, and high incidence (referred to as "other reason"). We chose to present all three curves because, in our view, a single curve focusing only on "economics" or "popularity" wouldn't adequately capture the interplay among these three factors as the lambda parameter changes. Showing both curves immediately clarifies for the reader the inverse relationship: as one factor gains importance, the other diminishes, highlighting the inherent symmetry. Also, as cited in the manuscript, the node popularity, unlike the economic size, while not prevailing in the reasons to form a connection, is always present in the linking probability (Eq. 2). For these* |

| | |
|---|---|
| | *reasons, in our opinion, it is appropriate to show all curves.* |
| **Minor points and suggestions** | |
| - the manuscript's writing could be improved, and some sentences should be streamlined to enhance the paper's overall readability and avoid ambiguities | Point taken. We've improved the manuscript in accordance with your suggestions, focusing on eliminating ambiguities, reducing repetition, and streamlining complex sentences to enhance overall clarity and readability. |
| - the Results section could be split into subsections dedicated to each experiment | Thank you for the suggestion. We break down the Results section into three subsections for each experiment (**Experiment 1, 2, 3**) that will definitely make it easier for readers to follow our findings. |
| - as they are the backbone of the contribution of this work, I think Eq (1) and Eq (2) in the Supplementary Information should be moved to the main text along with their explanation | We appreciate your feedback. Both equations (**Eq 1** and **Eq 2**) have now been moved into the main manuscript. |
